# The Positivity Bias Phenomenon in Face Perception Given Different Information on Ability

**DOI:** 10.3389/fpsyg.2017.00570

**Published:** 2017-04-27

**Authors:** Sasa Zhao, Yanhui Xiang, Jiushu Xie, Yanyan Ye, Tianfeng Li, Lei Mo

**Affiliations:** ^1^Center for the Study of Applied Psychology, School of Psychology, South China Normal UniversityGuangzhou, China; ^2^Guangdong Key Laboratory of Mental Health and Cognitive Science, South China Normal UniversityGuangzhou, China; ^3^Department of Psychology, Hunan Normal UniversityChangsha, China

**Keywords:** negativity bias, non-threatening information, positivity bias, facial perception, event-related potentials

## Abstract

The negativity bias has been shown in many fields, including in face processing. We assume that this bias stems from the potential threat inlayed in the stimuli (e.g., negative moral behaviors) in previous studies. In the present study, we conducted one behavioral and one event-related potentials (ERPs) experiments to test whether the positivity bias rather than negativity bias will arise when participants process information whose negative aspect involves no threat, i.e., the ability information. In both experiments, participants first completed a valence rating (negative-to-positive) of neutral facial expressions. Further, in the learning period, participants associated the neutral faces with high-ability, low-ability, or control sentences. Finally, participants rated these facial expressions again. Results of the behavioral experiment showed that compared with pre-learning, the expressions of the faces associated with high ability sentences were classified as more positive in the post-learning expression rating task, and the faces associated with low ability sentences were evaluated as more negative. Meanwhile, the change in the high-ability group was greater than that of the low-ability group. The ERP data showed that the faces associated with high-ability sentences elicited a larger early posterior negativity, an ERP component considered to reflect early sensory processing of the emotional stimuli, than the faces associated with control sentences. However, no such effect was found in faces associated with low-ability sentences. To conclude, high ability sentences exerted stronger influence on expression perception than did low ability ones. Thus, we found a positivity bias in this ability-related facial perceptual task. Our findings demonstrate an effect of valenced ability information on face perception, thereby adding to the evidence on the opinion that person-related knowledge can influence face processing. What’s more, the positivity bias in non-threatening surroundings increases scope for studies on processing bias.

## Introduction

Studies have shown that people display a preferential processing of negative information (e.g., negative facial expressions, immoral behaviors) than the corresponding positive one (Baumeister et al., 2001; Rozin and Royzman, 2001; Dyck et al., 2009). This phenomenon is known as the “negativity bias" and has been investigated within different domains, such as impressions formation, decision-making, social interaction, moral judgment, etc (Baumeister et al., 2001; Rozin and Royzman, 2001). Such a bias is also very common in face related studies: negative faces are preferentially processed; in terms of contextual influences on facial processing, negative information is always more influential than other information. However, presently, there are limited studies regarding the existence of a positivity bias. Therefore, this study aimed to explore the positivity bias in face processing.

The information processing bias toward negative stimuli may manifest in attention, memory, or perception. Studies show that negative faces draw more attention or are remembered better than are positive or neutral faces (Hansen and Hansen, 1988; Öhman et al., 2001; Anderson et al., 2011; Tsukiura et al., 2013). For example, Hansen and Hansen (1988) reported that the speed of identifying an angry face from a crowd of smiling faces was faster than vice versa. Tsukiura et al. (2013) found that the faces with an untrustworthy impression were remembered more accurately than those with a neutral or trustworthy impression were. Furthermore, studies show that the processing of human faces is affected not only by facial movements but also by context information (e.g., person-related information). The negativity bias exists in the context effect is conceptualized as stronger influence that negative information had on faces than did positive information (Abdel Rahman, 2011; Anderson et al., 2011; Baker et al., 2013; Suess et al., 2015; Luo et al., 2016). For instance, Anderson et al. (2011) explored the impact of gossip on the processing of neutral faces, with the paradigm of binocular rivalry. The result indicated that the neutral faces paired with negative gossip dominated in visual awareness significantly longer than did faces paired with other gossip. However, no difference was found for the faces paired with positive and neutral gossip. Baker et al. (2013) investigated the influence of moral behaviors on face recognition. They firstly presented vignettes related to moral behaviors (either immoral, morally neutral, or altruistic) with neutral faces, and then asked participants to identify the target faces within a set of faces with different levels of trustworthiness. Results showed that faces paired with immoral vignettes were recognized as less trustworthy than the actual faces, while, there was no difference in the altruistic or neutral condition, namely, only immoral behaviors influenced facial recognition memory. Crucially, by means of event-related potentials (ERP), the negative personal knowledge bias on the faces was also found in the early perceptual processing period (Abdel Rahman, 2011; Wieser et al., 2014; Suess et al., 2015). Suess et al. (2015) reported that the neutral expressions of unfamiliar faces paired with negative biographical information were perceived as more negative than the faces paired with relatively neutral information, indexed by larger early posterior negativity (EPN), but the effect was not apparent for positive biographical information. EPN was taken as the earliest ERP component reflecting valenced personal information influence on facial perception (Wieser et al., 2014; Luo et al., 2016).

However, it should be noted that the negative information involved in the previous studies mentioned above is mostly the one carrying threat, such as angry faces or evil behaviors (Öhman et al., 2001; Abdel Rahman, 2011; Feldmann-Wüstefeld et al., 2011). For security and survival, people prioritize paying attention to the potential threat in the environment and display the “negativity bias” in information processing. From the perspective of evolution, the threat-related negativity bias is reasonable and of high adaptive value: the consequences of dangerous stimuli are often much more dramatic than those of ignoring or reacting slowly to neutral or even appetitive stimuli. But what if there is no threat in the surroundings? Specifically, we were interested in whether negativity bias still exists when the negatively valenced information is not threatening or adverse (we define such information as “non-threatening information”).

The current study assumes that negative non-threatening information (e.g., unattractive faces, low ability information) does not pose a threat to our survival and security; thus, it would not make people go on alert. The corresponding positive one (e.g., attractive faces, high ability information), however, carries desirable, beneficial information. In such cases, the positive information may have a more powerful influence than the negative one. Some studies have shown indirect evidence for this assumption. Research showed that in aesthetic processing, compared with non-attractive faces, the attractive faces elicited an EPN (Werheid et al., 2007). The EPN is closely related to personal selective attention in the early phase (Schupp et al., 2007; Frühholz et al., 2011). A recent study also found that attractive faces dominated in visual awareness significantly longer than average and unattractive ones (Mo et al., 2016). However, none of them has directly explored the positivity bias or summarized the attribute of the negative information. Accordingly, combining behavioral assessment and ERP technology, we planned to test the “positivity bias” effect that non-threatening information may have on face perception.

We chose ability as a representative of non-threatening information in the present study. Ability is appropriate because low ability information carries no threat. At the same time, ability (or competence) is one of the universal dimensions of social cognition, playing an important role in person perception and evaluation (Skowronski and Carlston, 1987; Fiske et al., 2007; Freddi et al., 2014). Based on this, we further used a similar paradigm as those studies displaying the negativity bias, a minimal affective learning task (Abdel Rahman, 2011; Suess et al., 2015; Luo et al., 2016), for a direct comparison. In the current experimental tasks, the neutral expression faces were paired with high ability, low ability or control sentences, to test whether valenced ability information could bias the perception of facial expressions. If the expression ratings of the faces paired with high ability sentences show greater changes between pre- and post-learning than other faces (Experiment 1: Behavioral Assessment), and the EPN of the faces paired with high ability information is more pronounced (Experiment 2: ERP Data), we can conclude that there does exist a “positivity bias” evident in the effect of ability information on face perception.

## Experiment 1

### Method

#### Participants

Participants were recruited via target advertising on social media sites of South China Normal University. Thirty-three participants took part in the experiment for a small monetary compensation. One participant’s data was missing and another participant’s data was removed because his expression rating in the high ability group was out of ±3 SD in the post-learning. The remaining 31 participants (19 female) had a mean age of 20.74 years (*SD* = 2.14). All participants were right-handed and had normal or corrected-to-normal vision. None of them had any neurological impairment or had used psychoactive medication, nor had any of them participated in our other experiments. All participants gave their informed consent before the experiment. The current study was conducted under approval of the Academic Committee of the Department of Psychology at South China Normal University.

#### Design

The current study used a 2 (Learning: pre-learning, post-learning) × 3 (Ability: high, low, control) within-subjects design. The dependent variable was facial expression ratings.

#### Materials

##### Faces

Thirty-six unfamiliar gray-scale photos of male and female faces with neutral expressions were chosen from the Chinese Facial Affective Picture System (CFAPS; Gong et al., 2011). All photos were frontal headshots. Then, they were edited for homogeneity of all features (i.e., the hair, ears, neck, and so on were all removed; the size of the faces were scaled to 2.7 cm × 3.5 cm), using Photoshop CS 6.0.

##### Ability sentences

We selected 25 behaviors that could distinguish levels of individual ability and adapted each behavior to one of the three kinds of sentences: high ability, low ability, and control. For example, for the behavior related to “sales,” the high ability sentence was “Ranked first in sales many times,” the low ability sentence was “Failed to meet sales targets many times,” and the neutral sentence (which was not related to ability) was “Received sales target for this season.” A different group of 30 participants took part in the rating of these behavioral sentences according to the degree of ability on a 9-point scale (1 = *very low*, 9 = *very high*). Meanwhile, participants were asked to rate whether or not these behavioral sentences concerned ability (1 = *yes*, 0 = *no*). Based on the rating results, we chose 12 behaviors as the target materials (Mean ± SD ability ratings: high ability = 7.58 ± 0.36, low ability = 2.70 ± 0.29; the range of rating scores for high ability is 7.16 to 8.44, and the low ability is 2.12 to 3.18; Supplementary Material). A paired *t*-test indicated that the average ability level ratings between the high ability and low ability sentences differed significantly, *p* < 0.001.

##### Formal experimental materials

The 36 target faces were paired with the 36 ability sentences. These “face-sentence” pairs constituted our formal experimental materials.

#### Procedure

The experiment consisted of three phases: pre-learning, learning, and post-learning.

##### Pre-learning phase

Each trial began with a fixation cross displayed in the center of the screen for 500 ms, followed by a blank screen for 300 ms. Then, one of the 36 faces appeared following a random order. Participants were instructed to judge each face by rating the facial expression on a 9-point scale from very negative (1) to very positive (9), analogous to the Self-Assessment Manikin (Bradley and Lang, 1994). Each stimulus was displayed until the participant keyed his or her response.

##### Learning phase

In this phase, participants viewed face-sentence pairs. The faces appeared in the center of the screen, and the sentences appeared just below the faces. Participants were told to remember the pairings by imagining each person performing the behavior described in the corresponding sentence. Each of the 36 target faces was paired with a unique descriptive ability sentence that was high-ability, low-ability, or control (Each kind had the same total number). The three kinds of sentences were counterbalanced across participants. Different participants were shown different face-sentence pairs. The pairs were each displayed on the computer screen for 5000 ms with an 800 ms intertrial interval. Each face-sentence pair repeated five times in a random order, constituting a total of 180 experimental trials.

##### Post-learning phase

The procedure was the same as that in the pre-learning. A total of 36 faces appeared one at a time in a random order, and participants were asked to rate the facial expressions on a 9-point scale from very negative (1) to very positive (9). All of the faces were repeated twice.

### Results

**FIGURE 1:**
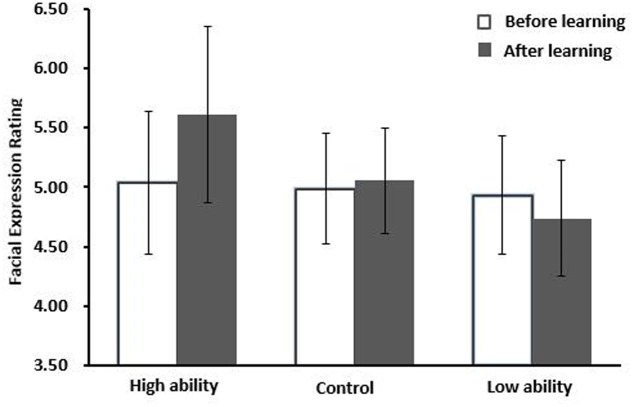
**Mean facial expression ratings (Mean ± SD) before and after the presentation of different kinds of ability information (high, low vs. control) for Experiment 1**.

Repeated measures analysis of variance (ANOVA) with the factors Ability Sentence (high, low, control) and Learning (pre-learning, post-learning) were carried out. There was a main effect of ability sentence, *F*_(2,60)_ = 14.18, *p* < 0.001, $\eta_{p}^{2}$ = 0.321, and a significant interaction of Ability Sentence × Learning, *F*_(2,60)_ = 16.64, *p* < 0.001, $\eta_{p}^{2}$ = 0.350. Simple effect analysis showed that faces associated with high ability information were rated as more positive in post-learning compared to pre-learning, *F*_(1,30)_ = 19.35, *p* < 0.001, $\eta_{p}^{2}$ = 0.392. Likewise, faces associated with low ability information were rated as more negative, *F*_(1,30)_ = 4.51, *p* = 0.042, $\eta_{p}^{2}$ = 0.131. Faces associated with control sentences did not differ between pre-learning and post-learning, *F*_(1,30)_ = 0.41, *p* = 0.530 (see **Figure 1**).

We further examined the difference between pre-learning and post-learning for the high ability condition compared to the low ability condition. The result showed that the difference for the high ability condition was larger than that of the low ability condition, *F*_(1,30)_ = 5.41, *p* = 0.027, $\eta_{p}^{2}$ = 0.153. As expected, these findings suggested “positivity bias” in facial perceptual processing for ability character information. As no differences emerged between pre-learning and post-learning for the control condition, we can exclude the mere exposure effect as a cause (Zajonc, 2001).

## Experiment 2

### Method

#### Participants

Eighteen participants took part in the experiment for a small monetary compensation. One outlier was excluded because of the abnormal reaction time (<200 ms). The remaining 17 participants (10 female) had a mean age of 22.59 years (*SD* = 1.62). All participants were right-handed and had normal or corrected-to-normal vision. None of them had any neurological impairment or had used psychoactive medication, nor had any of them participated in our other experiments. All participants gave their informed consent before the experiment. The current study was conducted under approval of the Academic Committee of the Department of Psychology at South China Normal University.

#### Design

The current study used a 2 (Learning: pre-learning, post-learning) × 3 (Ability Sentence: high, low, control) within-subjects design. The dependent variables were the facial expression ratings, N170 and EPN.

#### Materials

Experiment 2 used the same materials as Experiment 1.

#### Procedure

All participants were seated comfortably in a dimly lit, acoustically and electrically shielded room. Stimuli were presented using E-Prime1.1 at the center of a monitor that was placed at eye level 90 cm in front of the participants. The background of the screen was white, and the brightness, contrast, and color were all set consistently. Participants were instructed to take part in a memory experiment. As in Experiment 1, the procedure of Experiment 2 included three sessions: pre-learning, learning, and post-learning. The EEG test was only conducted during the post-learning.

##### Pre-learning Phase

A total of 36 faces appeared in a random order. When each face was presented on the screen, participants were instructed to rate the facial expressions (To adapt to the space on the monitor for EEG, we reduced the 9-point scale to 7-point scale, 1 = *very negative*, 7 = *very positive*).

##### Learning Phase

The learning phase was the same as in Experiment 1, except for two changes. In Experiment 2, each “face-sentence” pair was repeated four times, and to test whether participants had learnt the pairing, a memory test was added after learning. In the memory test, a face appeared in the center of the screen with two sentences below it. Participants had to indicate which of the two sentences described the correct behavior related to the face by pressing the ‘F’ or ‘J’ key on the keyboard with either their left or right index finger. The assignment of keys for indicating the correct answer was random across trials. All sentences and faces were the same as those in the learning task. Only participants who passed the memory test with higher than 80% accuracy continued to the next phase, and the others repeated the learning phase. All the participants could attain higher than 90% accuracy for a second time.

##### Post-learning phase

**FIGURE 2:**
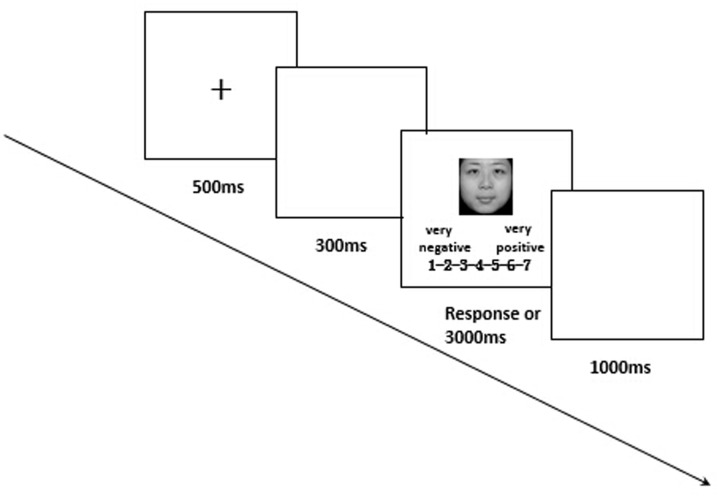
**Sequence of events in the post-learning**.

Each trial started with the presentation of a fixation cross for 500 ms, followed by a blank screen for 300 ms. The target face was then presented for 3000 ms or until the participant made his or her response (a 7-point facial expression rating, 1 = *very negative*, 7 = *very positive*). All faces were the same as those in the learning task. Participants were asked to concentrate on viewing the faces first before making their response. Participants’ electrical brain activity was collected during this stage. One second after the response, the next trial began (see **Figure 2**). The 36 faces repeated four times in a random order, and thus there were 144 trials in total.

#### EEG Recording and Data Analysis

The EEG was recorded with Ag/AgCl electrodes from 64 sites, according to the extended 10–20 system, at a sampling rate of 1000 Hz. Both the left mastoid and the right mastoid were used as the reference, and data were mathematically re-referenced off-line to an average reference. Both vertical (below the left eye) and horizontal (at the outer canthus of the right eye) electrooculograms were recorded. Electrode impedance was kept below 5 kΩ.

Off-line EEG analysis was performed with the computer software Brain Vision Analyzer Version 2.0 (Brain Products). EEGs were filtered using a 30 Hz low-pass and corrected for horizontal and vertical ocular artifacts. The remaining artifacts were eliminated with a semiautomatic artifact rejection procedure (amplitudes over ± 80 μV, changing more than 50 μV between samples). The EEG was segmented into epochs of 1.2 s, starting 200 ms prior to stimulus onset. According to the matched sentence, faces were divided into three groups: the high ability group, the low ability group, and the control group.

Research showed that faces elicit a clear negative deflection around 170 ms after stimulus onset; this negative peak is known as the N170 component (Bentin et al., 1996; Sagiv and Bentin, 2001). As N170 is particularly sensitive to faces, it is known as an index of an early structural processing of facial features and configurations (e.g., Bentin et al., 1996; Eimer, 2000). Numerous studies have shown that the EPN reflects facilitated capture of attentional resources, selective motivated attention, the evaluation of perceptual characteristics, and the selective processing of emotional stimuli (Schupp et al., 2003, 2007; Olofsson et al., 2008; Suess et al., 2015). Starting at around 150 ms, the EPN component is a relative negative deflection usually observed over temporo-parieto-occipital brain regions and is maximally pronounced around 260–280 ms after stimulus onset (Schupp et al., 2003, 2007; Abdel Rahman, 2011; Wieser et al., 2014). It is thought to reflect the mainly arousal-driven differential processing of emotional (compared to neutral) visual stimuli areas (Wieser et al., 2010). Specifically, emotional stimuli in comparison to neutral stimuli elicit larger EPN. According to the grand average, the three conditions began to diverge at nearly 230 ms in the temporo-parieto-occipital regions. Based on previous findings of early emotion effects in the EPN (Abdel Rahman, 2011; Klein et al., 2015; Luo et al., 2016), eight electrode sites for two ROIs were chosen for statistical analysis in the time window of 130–180 (N170) and 250–300 ms (EPN): CP5, P5, P7, PO7 (ROI: left posterior); CP6, P6, P8, PO8 (ROI: right posterior). Both of their amplitude differences were assessed with separate 2 (Ability Sentence: high, low, control) × 3 (Laterality: left, right) repeated-measures analyses of variance (ANOVAs). In all analyses, the Greenhouse–Geisser correction for non-sphericity was applied if Mauchly’s test of sphericity was significant.

### Results

#### Behavioral Results

A 2 (Learning: pre-learning, post-learning) × 3 (Ability Sentence: high, low, control) repeated-measures ANOVA yielded main effects of Ability Sentence, *F*(2,32) = 7.08, *p* = 0.007, $\eta_{p}^{2}$ = 0.307, and interactions of Learning and Ability Sentence, *F*(2,32) = 8.24, *p* = 0.004, $\eta_{p}^{2}$ = 0.340. A separate analysis for ability sentences revealed that faces associated with high ability information were perceived as more positive after learning than before learning, *F*(1,16) = 6.34, *p* = 0.023, $\eta_{p}^{2}$ = 0.284 (Mean ±*SD*: 3.96 ± 0.33; 4.33 ± 0.40). No difference was found for either the low ability condition or the control condition, *F*(1,16) = 0.38, *p* = 0.549 (Mean ±*SD*: 3.85 ± 0.44; 3.79 ± 0.44); *F*(1,16) = 0.03, *p* = 0.864 (Mean ±*SD*: 4.07 ± 0.43; 4.05 ± 0.40).

#### ERP Results

##### N170

**FIGURE 3:**
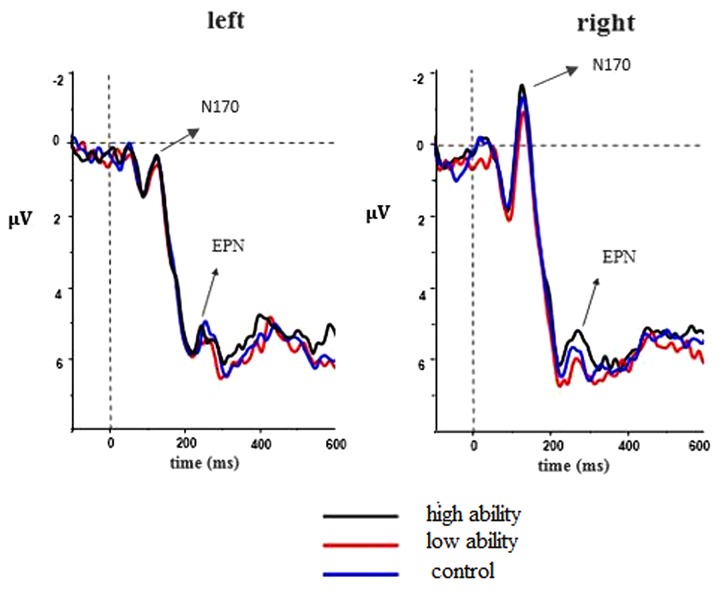
**Grand-averaged event-related potential (ERP) waveforms for high, low, and control ability conditions**. The ROI of left laterality includes CP5, P5, P7, PO7; and the corresponding right laterality includes CP6, P6, P8, PO8.

A two-factor (Ability Sentences: high, low, neutral; Laterality: left, right) repeated-measures ANOVA was conducted for the mean amplitude of N170. There was a significant main effect of Laterality, *F*_(1,16)_ = 7.53, *p* = 0.014, $\eta_{p}^{2}$ = 0.320, where over the right posterior sites a larger negativity was observed. However, main effect of Ability Sentence and interaction effect were not significant, *F*_(2,32)_ = 0.73, *p* = 0.442; *F*_(2,32)_ = 0.30, *p* = 0.742 (see **Figure 3**).

##### Early posterior negativity

The same repeated-measures ANOVA was conducted for the mean amplitude of EPN, just as N170. There was a significant main effect of Ability Sentence, *F*_(2,32)_ = 3.64, *p* = 0.045, $\eta_{p}^{2}$ = 0.185, and a significant interaction between Ability Sentence and Laterality, *F*_(2,32)_ = 5.43, *p* = 0.012, $\eta_{p}^{2}$ = 0.253. A simple effect analysis was conducted. For the left laterality, no difference was found for the comparison among ability conditions, *p*s > 0.1. For the right laterality, the three ability conditions differed significantly, *F*_(2,32)_ = 6.61, *p* = 0.006, $\eta_{p}^{2}$ = 0.292. Specifically, the high ability condition elicited a more pronounced negativity compared to the low ability condition, *F*_(1,16)_ = 16.20, *p* = 0.001, $\eta_{p}^{2}$ = 0.503, and the control condition, *F*_(1,16)_ = 8.00, *p* = 0.012, $\eta_{p}^{2}$ = 0.333. The difference between the low ability condition and the control condition failed to reach statistical significance, *F*_(1,16)_ = 0.51, *p* = 0.486 (see **Figure 3**).

Further, we’ve made a Pearson’s correlation test between facial expression ratings and EPN amplitudes: Pearson’s *r* = -0.376, *p* = 0.006. Results showed that the higher the ratings is, the larger the EPN amplitude is. The change trend of behavioral ratings is consistent with the counterpart of EPN amplitudes.

## Discussion

The present study investigated the influence of non-threatening information (i.e., ability information, whose negative dimension involves no threat) on human face processing. Specifically, neutral faces and valenced ability sentences (high ability, low ability, and control) were paired to identify whether the valenced ability information could bias the perception of neutral facial expressions. The effect was expected to be more evident after exposure to high ability sentences, displaying a positivity bias. Both behavioral and ERP data supported our hypothesis, i.e., compared with low ability behaviors, high ability behaviors induced stronger effect on people’s positivity/negativity ratings of faces.

In Experiment 1, the behavioral data showed a significant change in the expression ratings for the faces associated with both high ability and low ability (the high ability group were rated as more positive, while the low ability group were rated as more negative), but the change in the former was greater than those in the latter. The behavioral results in Experiment 2 were in accordance with the results of Experiment 1; greater change happened in the high ability group than in the low ability group after learning. In conclusion, the behavioral results suggested that the high ability information has a stronger effect on facial evaluation than the low ability information. The results of the ERP analyses in Experiment 2 showed that the three experimental conditions (high ability, low ability, and control) all elicited the obvious component of N170 and EPN in the time window 130–180 ms and 250–300 ms. For N170, we could not find any effect, which indicates that it may be unaffected by emotional faces (Eimer et al., 2003; Schacht and Sommer, 2009; Klein et al., 2015). For EPN, high ability elicited larger amplitude than other conditions, but no differences emerged between the low ability condition and control condition. Numerous studies have shown that EPN is related with more attention and enhanced perceptual encoding of emotional stimuli (Schupp et al., 2003, 2007; Abdel Rahman, 2011). Moreover, EPN is deemed as the earliest component reflecting the effect of affective information on facial perception (Wieser et al., 2014; Luo et al., 2016). In the present study, the EPN effect was more pronounced for faces paired with high ability sentences compared to those faces paired with low ability sentences. This suggests that the high ability information plays a more important role in expression perception and induces a larger bias in facial perception compared to low ability information. In other words, consistent with our hypothesis, the ability trait did exhibit a “positivity bias” influence in facial perceptual processing.

The valenced ability information effect found in our results provide new evidence that affective person-related information influences face processing (Wieser et al., 2014; Klein et al., 2015; Suess et al., 2015; Luo et al., 2016). This might have implications for social communications. Before meeting someone, we may have had some knowledge of them (e.g., ability information). Such available information may influence our inferences to their mental state or intentions, which further regulate our own behaviors and attitudes toward them. Further, the “positivity bias” results were different from those found in previous studies (Abdel Rahman, 2011; Suess et al., 2015; Luo et al., 2016). For example, Suess et al. (2015) paired morality-relevant actions with neutral faces in the learning phase. The results showed that a significant change in expression evaluation value occurred when the faces were paired with negative moral actions; by contrast, little change occurred when those faces were paired with positive moral actions. These results imply that in the process of perceptual impression formation, negative moral information is more influential; that is, there exists a negativity bias for moral information. Negative moral information involved in these studies contains threat; negative ability information in current study, however, is not threatening. Thus, combining our results with prior research on the positive attention bias (Werheid et al., 2007; Mo et al., 2016), we suggest the assertion that positive information may take precedence over negative one when people process non-threatening information whose negative aspect do not embody threat.

Comparing the present study with previous research results, we conclude that people tend to exhibit a “negativity bias” when they process information whose negative aspect carries survival threat; when it comes to information whose negative aspect carries no threat, people may tend to display a “positivity bias.” Such a processing style may be explained by human tendency to avoid harmful stimuli and approach beneficial stimuli, and it reflects the flexibility of humans’ cognitive processing (Smith et al., 2006; Rothermund et al., 2008). Out of basic survival needs, people focus on potential threats in their surroundings. The vigilance for dangerous signals can protect people from being hurt and is of great adaptive value for survival. However, in circumstances where there are no dangers, or where the potential threat is less than the potential benefits, the requirements for benefits are predominant. Thus, people focus more on favorable or potentially beneficial information. In this context, being sensitive to positive signals is of more adaptive value.

To summarize, our results add to the evidence on semantic context effects in face processing and suggest that not only threatening information (like morality information) but also non-threatening one (like ability information) can shape expression perception. What’s more, the “positivity bias” phenomenon has significant value for further understanding negativity bias in the facial perception, demonstrating that the “negativity bias” in face processing is not universal, but may be varied with the type of study stimuli. However, our study still has some limitations. Firstly, if an experiment testifying the negativity bias of threat-related information was added for a comparison, the results would be more conclusive. Secondly, we cannot ensure that low ability contains no threatening information absolutely in all cases, and further study should be conducted to explore this topic. Finally, further systematic studies can be carried out to strengthen our conclusion and enrich the effect, including experiments with other domains (like attention, memory) and other “non-threatening information (like social status information).”

## Ethics Statement

This study was approved by the Human Research Ethics Committee of South China Normal University. Informed written consent was obtained from participants before the experiment.

## Author Contributions

SZ: study design, data collection, data analysis, paper writing. YX: study design, paper writing. JX: data analysis, paper revising. YY: data collection, paper revising. TL: data collection, paper revising. LM: study design, paper writing.

## Conflict of Interest Statement

The authors declare that the research was conducted in the absence of any commercial or financial relationships that could be construed as a potential conflict of interest.
