# Supplementary material for: The Positivity Bias Phenomenon in Face Perception Given Different Information on Ability
Source: Front Psychol. 2017 Apr 27;8:570. doi: 10.3389/fpsyg.2017.00570 (PMC5407090; doi:10.3389/fpsyg.2017.00570)
Supplement: Supplementary file 1 [file Data_Sheet_1.DOCX]

**Appendix**

*Examples of language descriptions used in the present research*

| High Ability Description | Ranked first in sales many times. |
| --- | --- |
|  | Ranked first in many disciplines. |
|  | Published three best-seller books. |
|  | Received many offers from large companies. |
|  | Mastered five foreign languages skillfully.  Completed reading classical Chinese books easily. |
|  |  |
| Control Description | Received sales target for this season. |
|  | Took part in the final examination. |
|  | Copied the words from a book. |
|  | Planned to seek a job. |
|  | Planned to study English.  Opened a book. |
| Low Ability Description | Failed to meet sales target many times. |
|  | Failed to pass the exam in most of disciplines.  Had great difficulty in writing correct sentences. |
|  | Failed to get a job after graduating for three years. |
|  | Failed to express an English sentence after studying for a year. |
|  | Had great difficulty in reading any kinds of books. |
|  |  |
